# Torsion of a Theca-Lutein Cyst in Molar Pregnancy: A Rare but Critical Diagnosis

**DOI:** 10.7759/cureus.92956

**Published:** 2025-09-22

**Authors:** Adriana Bruno, Izabele P Jatobá, Marina N Soares, Carolina B Andrade

**Affiliations:** 1 Department of Gynecology and Obstetrics, Federal University of Bahia, Salvador, BRA; 2 Department of Gynecology and Obstetrics, Metropolitan Union for the Development of Education and Culture, Lauro de Freitas, BRA; 3 Department of Gynecology and Obstetrics, Bahiana School of Medicine and Public Health, Salvador, BRA

**Keywords:** acute abdomen, gestational trophoblastic disease, hydatidiform mole, molar pregnancy, ovarian necrosis, ovarian torsion, theca lutein cyst

## Abstract

We present the case of a 22-year-old woman who initially presented with vaginal bleeding following a two-month menstrual delay and ultrasound suspicion of an anembryonic pregnancy. She underwent two manual uterine evacuations at a regional hospital, and histopathology confirmed a complete hydatidiform mole. She was referred to a gestational trophoblastic disease reference center with severe pelvic pain and persistent vomiting for two days, refractory to analgesics. On admission, she exhibited signs of peritoneal irritation. Ultrasound revealed the presence of theca-lutein cysts in the left ovary and torsion of the right ovary. Emergency exploratory laparotomy confirmed adnexal torsion and necrosis, requiring right salpingo-oophorectomy. Postoperative recovery was favorable, and serum β-human chorionic gonadotropin (β-hCG) levels normalized after nine months of follow-up. This case highlights the importance of considering ovarian torsion as a possible complication in patients with molar pregnancy presenting with acute abdominal symptoms. Timely imaging is crucial for diagnosis and may allow conservative management, although adnexectomy remains necessary in cases of established necrosis. Awareness of this rare but serious complication is essential for prompt diagnosis and preservation of reproductive potential.

## Introduction

Gestational trophoblastic disease (GTD) encompasses a spectrum of disorders arising from abnormal placental trophoblastic cells and is characterized by the production of human chorionic gonadotropin (hCG) as a specific tumor marker [1]. The premalignant forms include complete and partial hydatidiform mole (HM), in addition to the atypical placental site nodule, which has recently been recognized as part of GTD [2]. The malignant forms are grouped under the term gestational trophoblastic neoplasia (GTN), which includes choriocarcinoma, placental site trophoblastic tumor, and epithelioid trophoblastic tumor [1,2].

HM is the most common form of GTD. The incidence of HM varies geographically, with rates below 1 per 1,000 pregnancies in developed countries but significantly higher in parts of Asia, Africa, and Central America [2].

Although considered benign, it may pose significant clinical risks due to its potential for progression to GTN, with an estimated risk of 15%-20% for complete moles and 0.5%-1% for partial moles, as well as the occurrence of acute complications, including potentially life-threatening obstetric events, and, in severe cases, maternal death [1-3].

Regardless of the setting, all cases require outpatient follow-up after uterine evacuation, with regular serum hCG monitoring for early detection of GTN [1]. Among the potential complications of HM, theca-lutein cysts warrant particular attention. They result from ovarian hyperstimulation triggered by elevated hCG levels and regress spontaneously with hormonal normalization. However, complications such as torsion or rupture may occur, requiring emergency surgical intervention [4,5].

This case contributes to the literature by documenting the rare complication of ovarian torsion after uterine evacuation of a molar pregnancy. It highlights the importance of heightened awareness not only for malignant progression but also for unexpected acute complications during follow-up.

## Case presentation

A 22-year-old woman, gravida 2, with no comorbidities or significant gynecologic history, presented with two months of amenorrhea followed by vaginal bleeding. Initial pelvic ultrasound suggested an anembryonic pregnancy. Expectant management was adopted, but she subsequently developed heavy vaginal bleeding, and her serum β-hCG was 156,603 mIU/mL. A repeat ultrasound demonstrated an enlarged uterus (834 cm³), a heterogeneous endometrium, and intrauterine echogenic material with multiple vesicular images, consistent with gestational trophoblastic disease (GTD). The ovaries were initially reported as normal in size. These examinations were performed in a regional hospital in the countryside, and the original images were not available for inclusion in this report.

She underwent uterine evacuation by manual vacuum aspiration (MVA), followed the next day by a repeat procedure due to persistent endometrial thickening. Histopathological examination confirmed a complete HM. The patient was subsequently referred to the GTD Reference Center in Bahia, Maternidade Climério de Oliveira, Federal University of Bahia (UFBA).

**Figure 1:**
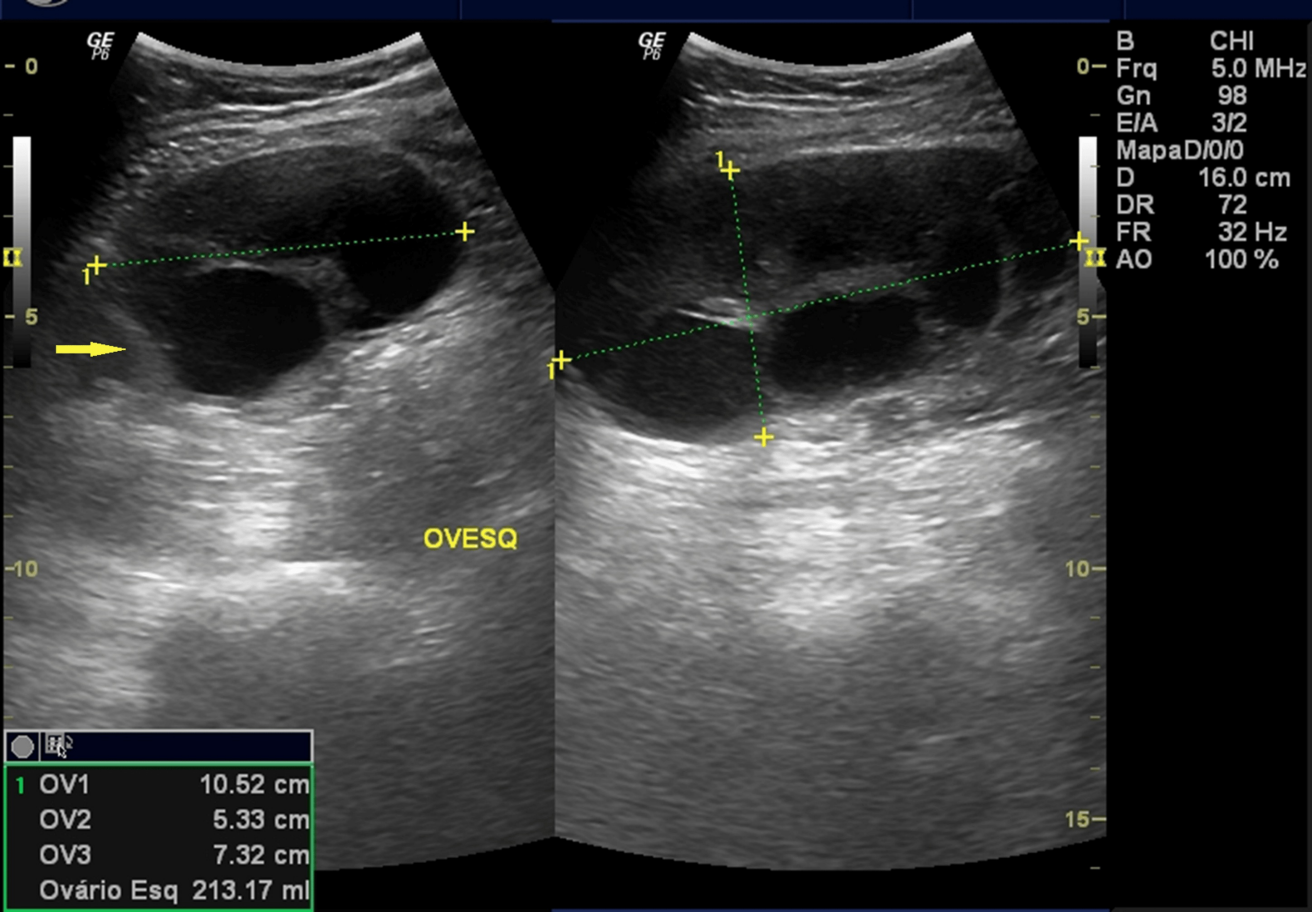
Left ovary with theca lutein cysts. Transvaginal ultrasound demonstrating an enlarged left ovary containing multiple theca-lutein cysts. The arrow indicates a representative cyst.

**Figure 2:**
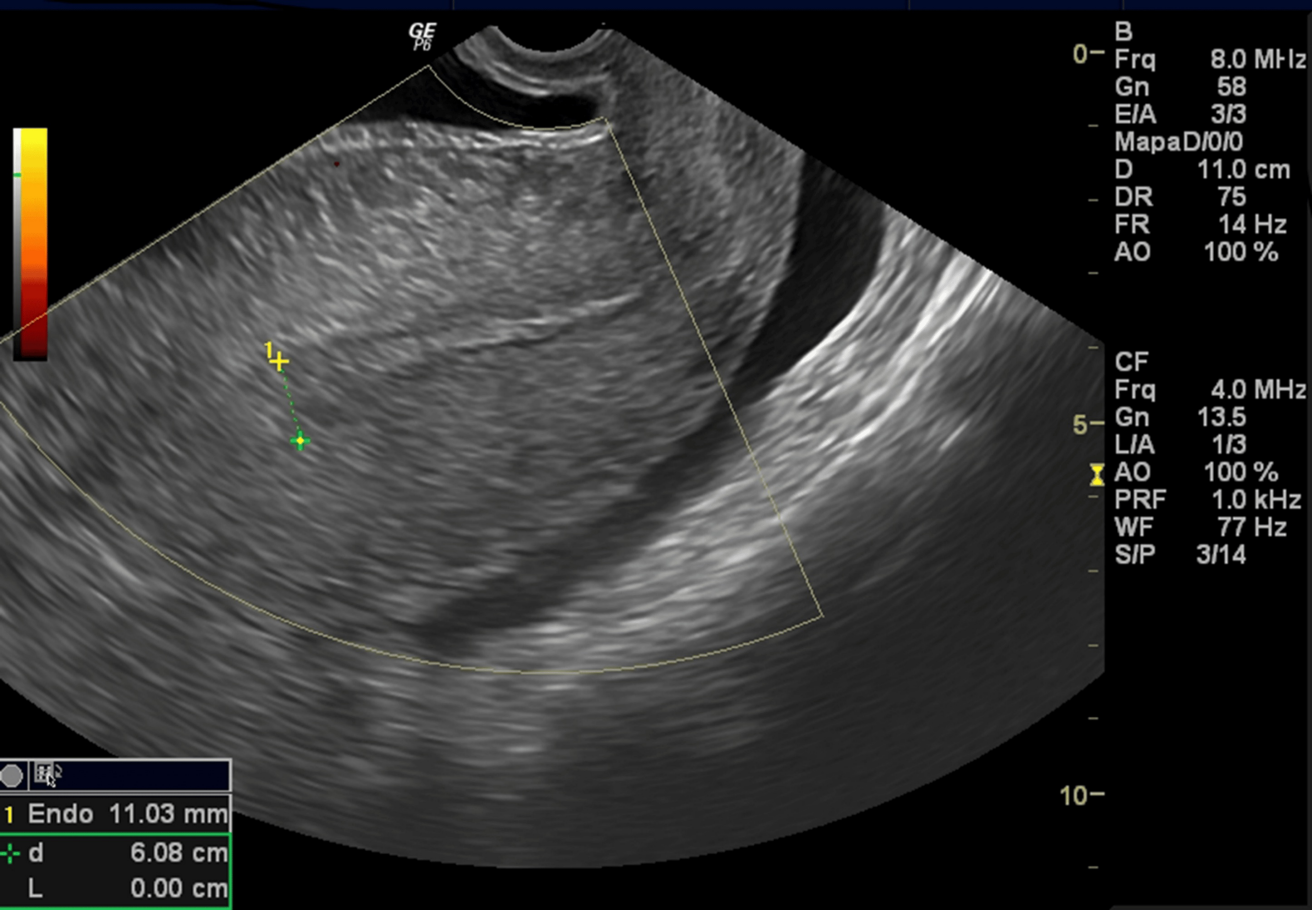
Uterus after evacuation. Transvaginal ultrasound showing the uterine cavity after evacuation, with residual heterogeneous endometrium measuring 11 mm by caliper.

**Figure 3:**
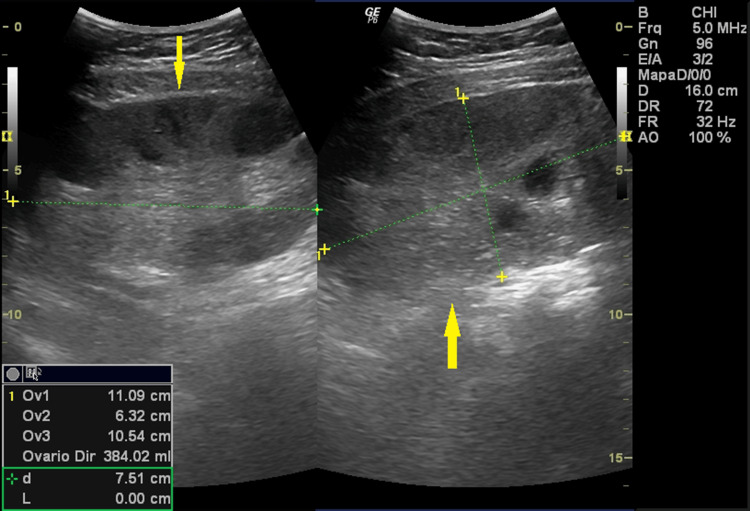
Right ovary with adnexal torsion. Transvaginal ultrasound with amplitude Doppler demonstrating an enlarged right ovary with heterogeneous parenchyma and absent vascular flow. The arrow indicates the ovary exhibiting sonographic features of torsion.

**Figure 4:**
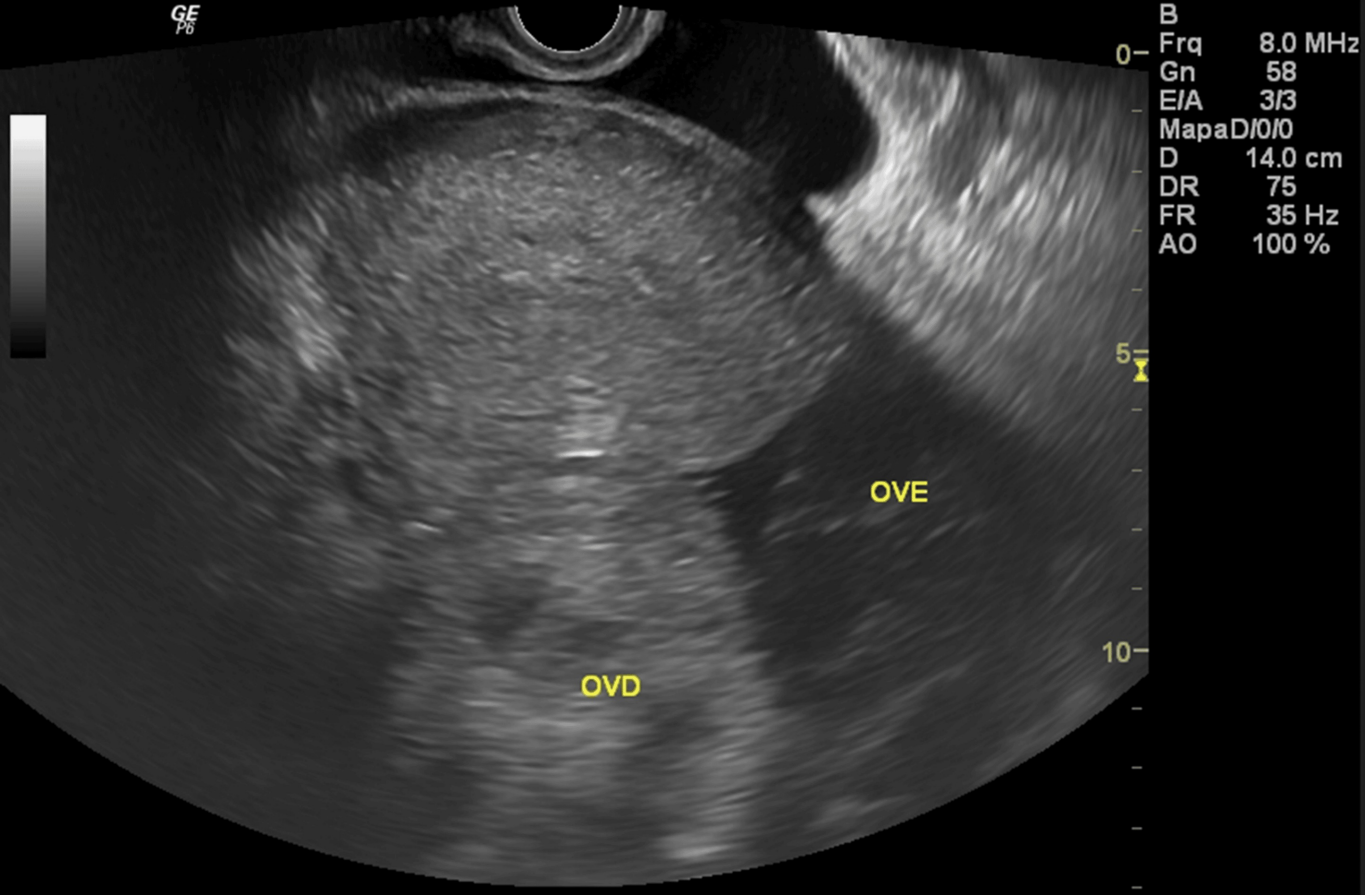
Comparative ultrasound of both ovaries. Transvaginal ultrasound showing the right ovary (OVD) enlarged, with increased parenchymal echogenicity, in contrast to the left ovary (OVE) with normal echogenicity.

At admission, five days after the first evacuation, she reported severe pelvic pain and vomiting for two days, refractory to analgesics. Physical examination revealed signs of peritoneal irritation. Transvaginal ultrasound showed the uterus after evacuation (Figure 1). The left ovary was enlarged with theca lutein cysts and preserved vascular flow (Figure 2), while the right ovary exhibited diffusely increased echogenicity, absent Doppler flow, and free pelvic fluid (Figure 3). A side-by-side comparison highlighted the difference between the vascularized left ovary and the avascular right ovary (Figure 4).

Laboratory investigations were within normal limits, including hematocrit (36.5%), hemoglobin (11.3 g/dL), leukocyte count (9.95 × 10⁹/L), thyroid-stimulating hormone (TSH, 0.04 µIU/mL), urea (18 mg/dL), creatinine (0.8 mg/dL), total bilirubin (0.2 mg/dL), aspartate aminotransferase (AST, 25 U/L), and alanine aminotransferase (ALT, 19 U/L). Although laboratory tests revealed a suppressed TSH level, the patient showed no clinical signs or symptoms of hyperthyroidism (Table 1).

**Table 1:** Laboratory results. All values were within normal limits according to standard reference ranges. TSH, thyroid-stimulating hormone; AST, aspartate aminotransferase; ALT, alanine aminotransferase

Test	Result	Reference range	Unit
Hematocrit	36,5	36-46	%
Hemoglobin	11,3	12-16	g/dL
Leukocyte count	9,95	4.0-10.0	×10⁹/L
TSH	0,04	0.4-4.0	µIU/mL
Urea	18	10-40	mg/dL
Creatinine	0,8	0.6-1.1	mg/dL
Total bilirubin	0,2	0.1-1.2	mg/dL
AST	25	10-40	U/L
ALT	19	7-56	U/L

**Figure 5:**
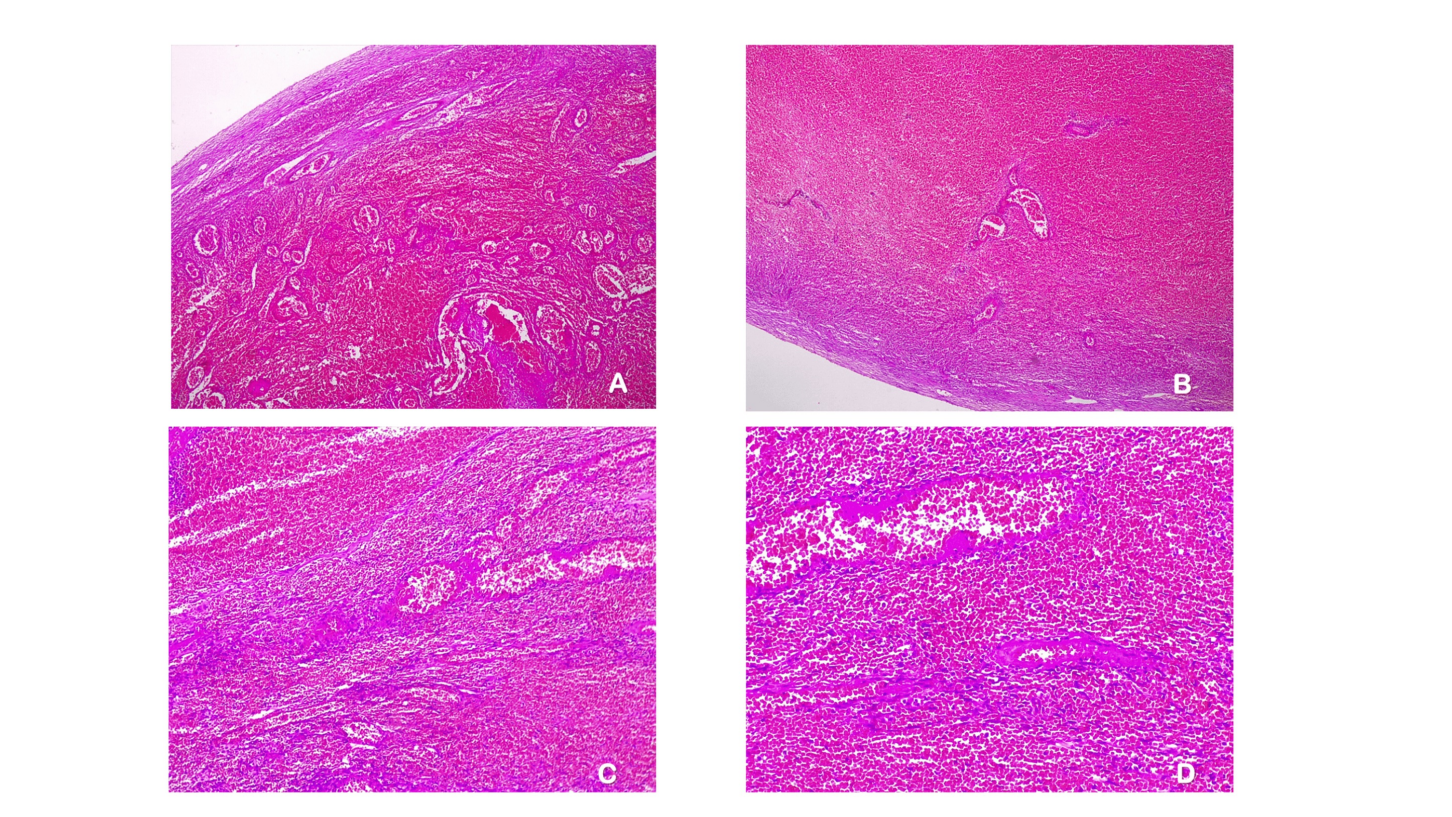
Histopathological findings of the right ovary with torsion. (A) Low-power view (4×) showing hemorrhagic infarction. (B) Low-power view (4×) of another area also demonstrating hemorrhagic infarction. (C) Intermediate magnification (10×) highlighting stromal involvement by hemorrhagic infarction. (D) High-power view (20×) revealing extensive hemorrhagic infarction with disruption of ovarian architecture.

Exploratory laparotomy confirmed right adnexal torsion with ovarian necrosis, requiring right salpingo-oophorectomy. The postoperative course was uneventful. Histopathology confirmed coagulative necrosis and parenchymal hemorrhage of ovarian tissue (Figure 5). 

Follow-up imaging revealed a persistent theca lutein cyst (83.5 cm³) in the left ovary with preserved vascular flow. The patient was discharged on postoperative day five with a serum β-hCG of 9,454 mIU/mL. She received oral contraceptives and counseling on the importance of strict follow-up. The left ovarian cyst regressed spontaneously, and β-hCG levels normalized within approximately nine months. Surveillance was continued monthly for six months after hormonal remission (β-hCG < 5 mIU/mL).

## Discussion

HMs are classified as complete HM (CHM) or partial HM (PHM), based on clinical presentation, histopathology, karyotype, and oncogenic potential. CHMs are usually associated with higher hCG levels than PHMs [2]. In this case, the elevated hCG levels, uterine enlargement, and characteristic ultrasound findings strongly suggested CHM, which was later confirmed histologically.

Although most molar pregnancies resolve with uterine evacuation, outpatient monitoring vigilance is required to detect GTN, which arises in 15-20% of CHMs [1-3]. In addition, acute complications such as hemorrhage, uterine perforation, hypertensive disorders, hyperthyroidism, and ovarian complications must be recognized and managed promptly.

Theca lutein cysts are prominent among such complications. These cysts are typically multilocular, bilateral, and self-limiting, resolving after hCG levels fall [6]. However, around 3% may evolve with torsion or rupture [4]. In this case, bilateral cysts were identified, one of which was complicated by ovarian torsion with subsequent necrosis.

Sudden-onset pelvic pain is the hallmark of ovarian torsion, often accompanied by nausea and vomiting. Although symptoms typically arise within 24 hours, delays in diagnosis increase the risk of adnexectomy. In our patient, symptoms persisted for over 48 hours, and surgical exploration confirmed right-sided torsion with necrosis, necessitating adnexectomy. Additional signs such as tenderness, tachycardia, and occasionally a palpable mass may also be present [5,7,8].

Cases of adnexal torsion associated with HM are extremely rare, with only a few reports described in the literature. For instance, Özdemir et al. reported bilateral adnexal torsion due to a postmenopausal HM, highlighting the severity of this complication even in unusual patient populations [9]. Similarly, Escobar-Ponce et al. described bilateral ovarian torsion following molar pregnancy in a young woman, emphasizing the need for prompt recognition and management to preserve patient outcomes [10]. These cases, along with ours, contribute to the limited body of knowledge on this uncommon but critical condition.

Our case highlights the importance of considering torsion in patients with molar pregnancies who return with nonspecific abdominal complaints. Right-sided torsion, as occurred in our case, is more frequent, potentially due to the longer utero-ovarian ligament and the relative mobility of adjacent intestinal loops, while the left side may be somewhat protected by the sigmoid colon [7].

Diagnosis of adnexal torsion requires an integrated clinical approach. Ultrasound evaluation with Doppler is the first-line modality, though its sensitivity ranges from 46% to 75%. MRI or CT typically provides similar findings and does not offer a significant diagnostic advantage over ultrasound [11]. In this case, ultrasonographic evaluation confirmed the clinical suspicion, supporting the decision for prompt surgical management. Crucially, the absence of classic sonographic signs does not exclude torsion, and surgical intervention should not be delayed when clinical suspicion is high.

Management of theca lutein cysts is usually conservative. Surgery is reserved for cases with complications such as torsion, rupture, or hemorrhage. When torsion occurs, conservative approaches like detorsion with or without cyst aspiration should be attempted if the adnexa are viable. Definitive treatment, such as adnexectomy, is reserved for nonviable tissue [5,7]. In the present report, adnexectomy was required because the adnexa were not viable at the time of surgery.

Duration of symptoms appears to be a more reliable predictor of necrosis than the macroscopic appearance of the ovary. The likelihood of preserving viable ovarian tissue is significantly reduced when torsion persists for 48 hours or more [12]. In our patient, the prolonged duration of symptoms may be associated with the intraoperative finding of necrosis, which precluded conservative management. In contrast, the contralateral ovary, although presenting with a theca-lutein cyst, maintained adequate vascularization, allowing preservation of hormonal function and reproductive potential.

Although laparoscopy is the preferred approach for managing adnexal torsion [7,8], laparotomy was performed in this case, as it was the only available option in this setting. Importantly, in settings where laparoscopy is unavailable, surgical intervention should not be delayed, as timely management is critical to preserving patient outcomes. In this case, management in a specialized center provided structured follow-up and timely intervention, which are considered essential in rare GTD-related complications [13].

## Conclusions

This case highlights the importance of vigilant monitoring during the post-molar period, especially in patients presenting with theca lutein cysts. Although adnexal torsion following molar evacuation is rare, it should be promptly considered in the differential diagnosis of acute abdomen due to its potential impact on ovarian viability. Clinicians should maintain a high index of suspicion when patients with molar pregnancy present with acute pelvic pain associated with nausea and vomiting. Timely imaging, such as Doppler ultrasound, is essential for diagnosis.

This report contributes to the limited literature on this complication and emphasizes the value of GTD reference centers in achieving accurate diagnosis and management. Furthermore, given that adnexectomy may compromise future fertility, conservative surgical strategies should be considered whenever feasible.

Early recognition and appropriate management can optimize outcomes and preserve reproductive potential in this vulnerable patient population.
